# How does pulmonary function impact QoL in patients with locally advanced NSCLC treated with chemoradiotherapy and durvalumab?

**DOI:** 10.2340/1651-226X.2026.45040

**Published:** 2026-03-19

**Authors:** Frigg Å. Sommervoll, Henrik Horndalsveen, Dag Einar Sommervoll, Jussi Koivunen, Tarje Onsøien Halvorsen, Bjørn Henning Grønberg, Marianne Aanerud, Saulius Cicenas, Nina Helbekkmo, Jarkko Ahvonen, Maria Silvoniemi, Gina Barrera, Maria M. Bjaanæs, Vilde D. Haakensen, Åsa Öjlert, Kersti Oselin, Åslaug Helland, Tesfaye Madebo

**Affiliations:** a Department of Clinical Science, University of Bergen, Bergen, Norway; b Department of Cancer Genetics, Institute for Cancer Research, Oslo University Hospital, Oslo, Norway; c Department of Oncology, Oslo University Hospital, Oslo, Norway; d Department of Clinical Medicine, University of Oslo, Oslo, Norway; e NMBU, Ås, Norway; f Department of Oncology and Radiotherapy, Oulu University Hospital, Oulu, Finland; g Medical Research Center Oulu, Oulu, Finland; h Department of Clinical and Molecular Medicine, NTNU, Norwegian University of Science and Technology, Trondheim, Norway; i Department of Oncology, St. Olavs Hospital, Trondheim University Hospital, Trondheim, Norway; j Department of Thoracic Medicine, Haukeland University Hospital, Bergen, Norway; k Department of Thoracic Surgery and Oncology, National Cancer Center, Affiliate of Vilnius University Hospital Santaros Klinikos, Vilnius, Lithuania; l Department of Pulmonology, University Hospital of North Norway, Tromsø, Norway; m Tays Cancer Center, Department of Oncology, Tampere University Hospital, Tampere, Finland; n Department of Pulmonary Medicine, Turku University Hospital, Turku, Finland; o Department of Pulmonology, Stavanger University Hospital, Stavanger, Norway; p Oncology and Haematology Clinic, North Estonia Medical Centre, Tallinn, Estonia

**Keywords:** Cancer, NSCLC, quality of life, COPD, lung

## Abstract

**Background:**

Impaired pulmonary function is common among patients with lung cancer and may negatively affect health-related quality of life (HRQoL). The primary objective of the present sub-study of the DART-trial was to assess the overall quality of life changes during treatment and stratified by the presence of Chronic Obstructive Pulmonary Disease (COPD).

**Methods:**

The investigator-initiated DART trial (NCT04392505) included patients with unresectable stage III non-small cell lung cancer (NSCLC) treated with chemoradiotherapy followed by durvalumab. Baseline pulmonary function was measured by spirometry, and patients were stratified by FEV1/FVC <70% (COPD) or ≥70% (non-COPD). HRQoL was assessed regularly using the EORTC QLQ-C30 and QLQ-LC13 questionnaires at screening and during treatment. A difference in mean score of ≥10 was defined as clinically significant.

**Results:**

A total of 86 patients initiated durvalumab and completed at least one HRQoL assessment; pulmonary function data were available for 64 patients. For the overall cohort, quality of life scores remained stable throughout treatment. Patients with COPD consistently reported lower global health scores than those with preserved lung function. The global health score among patients with COPD was not significantly different at end of treatment compared to baseline, however significantly lower than patients without COPD. Symptom trajectories across QLQ-C30 scales were stable in both groups. Dyspnoea was more prevalent among patients with COPD. In the LC13 module, no clinically significant differences were observed except for dyspnoea, which was consistently higher among patients with COPD.

**Interpretation:**

The HRQoL remained stable during chemoradiotherapy and durvalumab treatment in stage III NSCLC patients. Impaired lung function was associated with modestly lower HRQoL, though larger studies are needed to confirm subgroup effects.

## Introduction

Lung cancer is one of the most common malignancies and the leading cause of cancer-related mortality worldwide [1]. In Norway, 3435 new cases were diagnosed in 2024, and 2243 patients died from the disease (Cancer Registry of Norway [https://www.fhi.no/contentassets/1d3cf9facb9747a1b9148cb23a7f7c54/cancer-in-norway-2024.pdf]). Approximately 20–30% of patients with non-small cell lung cancer (NSCLC) present with stage III, locally advanced disease [2].

For patients with good performance status and unresectable stage III NSCLC without genetic alterations in the genes Epidermal Growth Factor Receptor (EGFR) or Anaplastic Lymphoma Kinase (ALK), the standard of care is radiotherapy given concurrently with platinum-based doublet chemotherapy (chemoradiation), followed by up to 1 year of durvalumab in patients with tumours expressing the protein Programmed Death -Ligand 1 (PD-L1) [3]. This approach, modelled by the PACIFIC trial, achieves cure in a subset of patients, however, the median progression-free survival is limited, and only 42% of patients are alive at 5 years [4, 5].

Approximately 40–70% of NSCLC patients also suffer from Chronic Obstructive Pulmonary Disease (COPD) [6–8]. The two diseases share major risk factors such as smoking, environmental pollutants, and occupational carcinogen exposure. Proposed mechanisms linking COPD and lung cancer include genetic predisposition, epigenetic regulation, and chronic local and systemic inflammation [9].

The COPD has been identified as an independent risk factor for mortality in stage III NSCLC, particularly in squamous cell carcinoma [10–12], although some studies report conflicting results [13]. Nevertheless, it’s important to note that standard eligibility criteria for concurrent chemotherapy and radiotherapy trials in locally advanced NSCLC typically exclude individuals with advanced COPD and cardiac comorbidities. In addition, tobacco smoking in cancer patients is associated with increased treatment toxicity, higher risk of a treatment failure, and poorer quality of life [14].

The coexistence of COPD complicates diagnosis and management of NSCLC. Overlapping symptoms may delay diagnosis or lead to misinterpretation [15]. Radiotherapy and immunotherapy can further impair pulmonary function due to inflammation, pneumonitis, or fibrosis [16–19]. Baseline pulmonary function parameters, such as Forced Exporatory Volume in 1 Second (FEV1), Forced Vital Capacity (FVC and Diffusing Capasity of the Lungs for Carbon Monoxide (DLCO), may predict risk of radiation pneumonitis [20].

While survival remains a central outcome, health-related quality of life (HRQoL) is increasingly recognized as a critical factor in treatment planning, especially given the substantial symptom burden experienced by these patients, including fatigue, pain, and impaired physical and social functioning. Two systematic reviews – Reale et al. and Marandino et al. – highlight persistent deficiencies in the reporting and integration of HRQoL data in phase III oncology trials [21, 22]. Despite growing awareness, HRQoL outcomes are often under-reported, delayed or omitted, limiting their influence on clinical decision-making [2]. Moreover, the impact of baseline pulmonary function on HRQoL during curative-intent therapy for stage III NSCLC remains poorly understood and there is a need for further studies [22, 23].

In the Durvalumab after RadioTherapy Trial (the Dart Trial), patient well-being is monitored every 6–12 weeks using two validated instruments: the European Organisaton For Research and Treatment of Cancer (EORTC) Quality of Life Questionnaire - 30 (QLQ-C30) (version 3) and the lung cancer-specific QLQ-LC13, capturing physical, emotional, and social dimensions. Here, we report changes in symptoms and functioning according to pre-treatment pulmonary function.

## Material and methods

### The DART-study

The DART-study is an open-label, multinational, investigator-initiated phase 2 trial aiming to identify biomarkers for stratifying treatment for patients with locally advanced non-small cell lung cancer (NSCLC). The study was conducted at 10 hospitals in Norway, Finland, Lithuania, and Estonia. Inclusion started on May 2020 and ended on December 2023. Patients with unresectable stage III NSCLC were enrolled and treated with curatively intended chemoradiotherapy (CRT), consisting of two cycles of platinum-based doublet chemotherapy every 3 weeks and radiotherapy 60–66 Gy in 30–33 fractions. Patients without disease progression following CRT received durvalumab 1500 mg every 4 weeks, preferably starting within 5 weeks of CRT completion, and continued until progression, intolerable toxicity, or a maximum duration of 12 months independent of PDL1 expression. Participants not starting durvalumab were excluded from further analyses. The follow-up includes a safety follow-up for up to 5 years, and a survival follow-up for up to a total of 10 years. Approval was granted by the Regional Committee for Medical and Health Research Ethics (reference 48665, November 28, 2019). All participants provided written informed consent. The trial is registered at ClinicalTrials.gov (NCT04392505). The primary endpoint in the DART-study was to determine how tumour mutational burden affected hazard ratio, with several secondary and exploratory endpoints, including the HRQoL analyses. The study was powered for the primary endpoint, and no estimation of power for the HRQoL analyses was performed. No specific inclusion requirements were defined regarding lung function, as this was a pragmatic trial with inclusion following clinical practice. For the analyses of HRQoL, we included patients completing at least one questionnaire at screening, and for the analyses related to lung function, we included patients with baseline measurement of lung function.

### Quality of life measurements

HRQoL was assessed using the EORTC QLQ-C30 (Version 3) and the lung cancer module LC13 from the screening timepoint (before chemoradiation) and at cycle 3 (6 weeks) and thereafter every 12 weeks during durvalumab treatment. These instruments enable a thorough assessment of the patients’ quality of life, encompassing physical, emotional, and social dimensions. QLQ-C30 is a general questionnaire for cancer patients, while QLQ-LC13 incorporates dimensions highly relevant for lung cancer patients.

The primary objective of this study was to assess the overall quality of life changes during treatment and stratified by the presence of COPD. Through this approach, the DART study aims to gather nuanced insights into the experiences and needs of lung cancer patients with clinically relevant comorbidity. The baseline was at inclusion in the study, before starting treatment.

### Pulmonary function

Pulmonary function tests, by spirometry, were conducted in adherence to the guidelines outlined by the American Thoracic Society/European Respiratory Society (ATS/ERS) and local standards for participants in the study, prior to study treatment.

Patients were classified according to the severity of airway limitation, as outlined by the latest Global Initiative for Chronic Obstructive Lung Disease (GOLD) strategy [24]. Specifically, individuals with a postbronchodilator FEV1/FVC ratio below 70% as assessed before starting chemoradiation, were identified as having COPD.

### Statistical analysis

Scores for the QLQ-C30 and QLQ-LC13 questionnaires were calculated according to the EORTC Scoring Manual, with raw scores standardized by linear transformation to range from 0 to 100 [25]. A higher score represents a better level of functioning and global health status/QoL or greater symptom severity. Clinically meaningful changes were defined as an absolute change (increase or decrease) in score from baseline of ≥10 points [26]. The following items were analysed: Global Health, Emotional functioning, Fatigue, Pain, Dyspnoea, from the QLQ-C30, and Arm/Shoulder pain, Chest pain, Other pain, Cough, Hemoptysis, Dyspnoea from the QLQ-LC13. We have used data from baseline and at cycle 3, 6, 9, and 12 of durvalumab. Scores from patients with and without COPD were compared. Data were reported using descriptive statistics with percentages, means, and standard deviation. We did not perform imputations of missing data. The results are given as the mean values ± one standard deviation unless otherwise stated. R (version 4.5.1) was used to perform calculations and produce graphical figures.

## Results

### Patients

**Figure 1:**
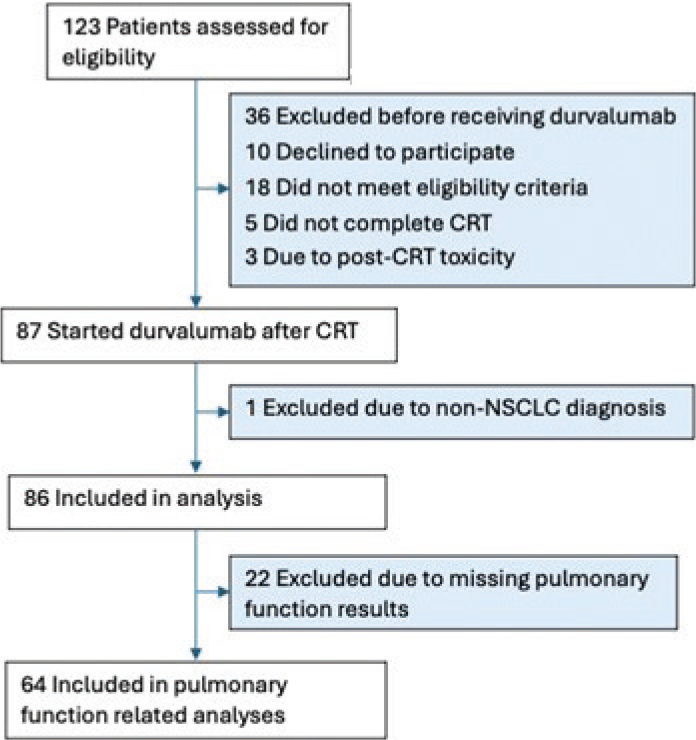
Flowchart.

Of the 123 patients included in the DART-trial, 86 NSCLC patients started treatment with durvalumab. All 86 completed at least one QoL questionnaire and were included in the present analyses. Figure 1 shows a study flowchart illustrating patient enrolment, exclusions, and pulmonary function test availability.

Characteristics of the 86 patients are shown in Table 1. The study cohort consisted of 52 men (60.5%) and 34 women (39.5%), with a median age of 69 years (range 36–85). Pulmonary function was recorded for 64 patients (74.4%) (Table 1), of whom 40 (62.5%) of the patients had FEV1/FVC < 70%, and 24 (37.5%) had FEV1/FVC ≥ 70%. The patients receiving durvalumab received a median of 13 cycles (average 10 cycles). The median Progression Free Survival (PFS) was 18.9 months.

**Table 1:** Clinical characteristics.

Clinical characteristics	*N* = 86 (all)	*N* = 40 (COPD)	*N* = 24 (no-COPD)
**Age: median (range)**	69 (36–85)	70 (51–80)	67.5 (51–85)
**Sex**			
Male	52 (60.5%)	22 (55%)	15 (62.5%)
Female	34 (39.5%)	18 (45%)	9 (37.5%)
**Smoking**			
Current	26 (30.2%)	13 (32.5%)	4 (16.7%)
Former	56 (65.1%)	27 (67.5%)	18 (75%)
Never	4 (4.7%)	0 (0%)	2 (8.3%)
**Performance status**			
0	34 (39.5%)	11 (27.5%)	12 (50%)
1	52 (60.5%)	29 (72.5%)	12 (50%)
**Histology**			
Adenocarcinoma	31 (36.0%)	12 (30%)	13 (54.2%)
Squamous cell carcinoma	49 (57.0%)	25 (62.5%)	7 (29.2%)
NSCLC NOS	6 (7.0%)	3 (7.5%)	4 (16.7%)
**PD-L1 expression**			
Negative (< 1%)	35 (40.7%)	17 (42.5%)	10 (41.2%)
Positive (≥ 1%)	51 (59.3%)	23 (57.5%)	14 (58.3%)
**Pulmonary function**			
FEV1/FVC < 70%	40 (62.5%)		
FEV1/FVC ≥ 70%	24 (37.5%)		

COPD: Chronic Obstructive Pulmonary Disease; NSCLC: non-small cell lung cancer; NOS: Not Otherwise Specified.

### Baseline Patient Recorded Outcome Measurements (PROMs)

An overview of baseline patient-reported outcome scores stratified by pulmonary function is presented in Table 2. Patients with FEV1/FVC < 0.7 reported lower global health and some symptoms were more severe compared to those with FEV1/FVC ≥ 0.7. Specifically, patients with reduced lung function (FEV1/FVC < 0.7) reported numerically lower (but not significantly different) scores on global health (60.63 ± 18.10 vs. 66.67 ± 17.92) and reported higher levels of dyspnoea (45.83 ± 28.93 vs. 29.17 ± 28.34) in the EORTC QLQ-C30 symptom scale (v3). The emotional function was not significantly different in the two groups. There were no significant differences in the baseline scores as measured by EORTC QLQ-LC13 between patients with or without COPD.

**Table 2:** PROMs at baseline.

	All patients (*N* = 86)		Patients (*N* = 40) FEV1/FVC < 0.7		Patients (*N* = 24) FEV1/FVC ≥ 0.7	
	*N*	Score	*N*	Score	*N*	Score
EORTC QLQ-C30 function scale^a^						
Emotional function	83	78.51 ± 20.75	40	78.54 ± 19.60	22	80.30 ± 17.92
Global Health	83	61.95 ± 20.04	40	60.63 ± 18.10	23	66.67 ± 24.10
EORTC QLQ-C30 symptom scale^b^						
Fatigue	82	31.03 ± 24.43	38	36.26 ± 24.60	23	22.22 ± 21.19
Pain	83	16.67 ± 24.00	40	20.83 ± 25.81	23	10.87 ± 19.85
Dyspnoea	86	34.50 ± 28.67	40	45.83 ±28.93	24	29.17 ± 28.34
EORTC QLQ-LC13 symptom scale^b^						
Arm / shoulder pain	86	14.73 ± 25.87	40	15.00 ± 26.09	24	15.28 ± 27.77
Chest pain	85	15.29 ± 23.32	40	14.17 ± 23.74	23	13.04 ± 19.43
Other pain	84	12.30 ± 20.55	39	11.11 ± 20.71	24	13.89 ± 21.80
Cough	86	45.35 ± 26.53	40	47.50 ± 28.13	24	41.67 ± 24.47
Hemoptysis	86	9.30 ± 20.86	40	12.50 ± 24.68	24	9.72 ± 20.80
Dyspnoea	86	26.87 ± 21.04	40	34.44 ± 23.84	24	18.06 ± 15.99

*N* = number of patients. Scores are presented as mean value ± standard deviation.

a Higher score indicates higher functioning (scale 0–100),

b Lower score indicates milder symptoms (scale 0–100).

### EORTC QLQ-C30 function scale

**Figure 2:**
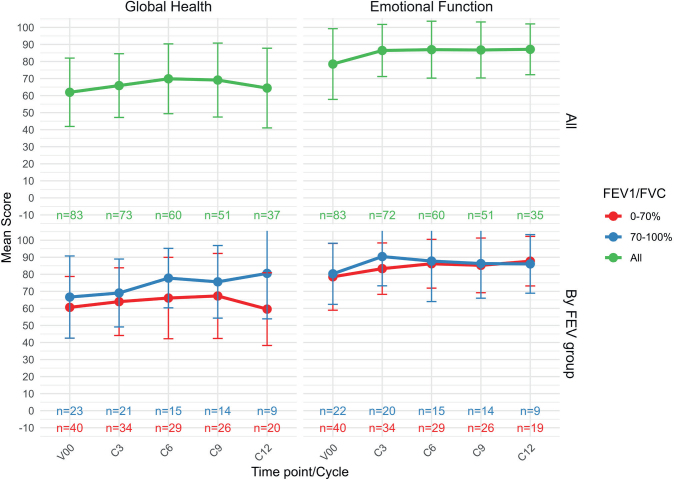
(a) Mean global health function (± SD) for all patients who initiated durvalumab treatment, across five time points (V00, (screening), C3 (cycle 3), C6 (cycle 6), C9 (cycle 9), C12 (cycle 12)). N* = Number of patients still on treatment. (b) Mean emotional function scores (± SD) for all patients who initiated durvalumab treatment, across five time points (V00, C3, C6, C9, C12). (c) Mean scores for global health for patients treated with durvalumab stratified by FEV1/FVC < or ≥ 0.7 (*N* = 64). (d) Mean scores for emotional function for patients treated with durvalumab stratified by FEV1/FVC group (*N* = 64).

The mean overall global health (± SD) for all patients who initiated durvalumab treatment, across five time points (V00 (Screening), and cycle (C) C3, C6, C9, C12) are shown in Figure 2a. Overall, global health scores remained relatively stable across treatment cycles, with mean values consistently around 60–70. A slight increase was observed around C6, but this was not sustained, and the overall trajectory suggests no significant decline in perceived health status over time.

Figure 2b shows the mean emotional function (± SD) for all patients who initiated durvalumab treatment, revealing a slight non-significant increase in emotional function during the treatment period.

Figure 2c shows mean global health scores stratified by pulmonary function (± SD). Overall, patients without COPD reported higher global health scores than those with COPD (approximately 6–20 points higher). Over the study period, mean global health scores showed a modest but steady increase among patients without COPD whereas those with COPD exhibited a slight initial improvement followed by a decline at the final assessment. Consequently, the difference between the two groups became more pronounced over time (Figure 2c). Importantly, there was no indication of a significant progressive decline in either group during treatment. Figure 2d shows that the emotional function increases non-significantly in both groups.

### EORTC QLQ-C30 symptom scale

**Figure 3:**
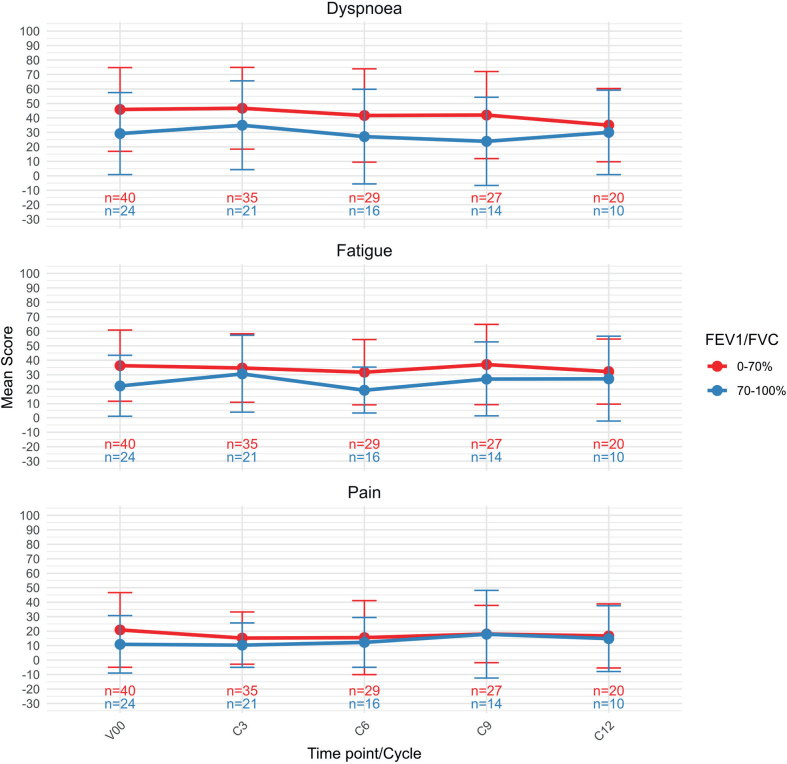
EORTC QLQ-C30 symptoms. A higher score represents more symptoms / higher burden. V00 = screening, C3 = cycle 3, C6 = cycle 6, C9 = cycle 9, C12 = cycle 12.

When stratified by pulmonary function, most QLQ-C30 symptom scores remained broadly stable during treatment, as shown in Figure 3. No clinically significant changes were observed for dyspnoea, fatigue or pain, in either groups, with both groups displaying stable trajectories across treatment cycles. Symptom scores for both fatigues, pain and dyspnoea (QLQ-C30 v3) were consistently higher in the impaired lung function group as compared to the patients with no COPD, although the difference diminished over time for all three symptoms over time.

### EORTC QLQ-LC13

**Figure 4:**
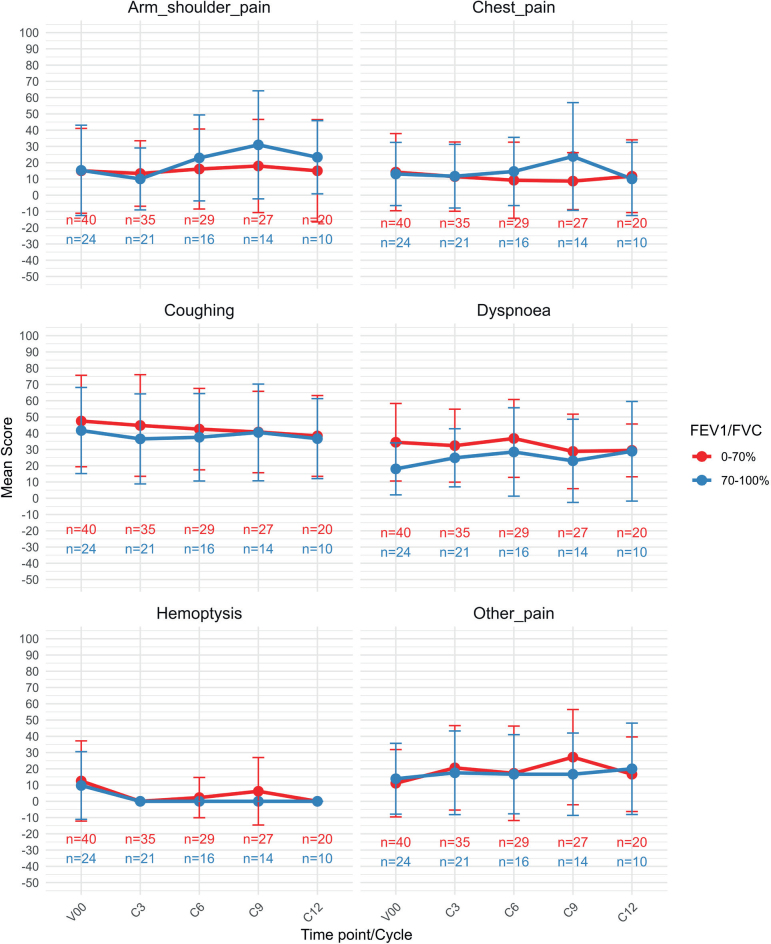
EORTC QLQ-LC13 symptoms. A higher score represents more symptoms / higher burden. V00 = screening, C3 = cycle 3, C6 = cycle 6, C9 = cycle 9, C12 = cycle 12.

Lung cancer specific symptoms were assessed thought EORTC QLQ-LC13. As shown in Figure 4, we see mean scores for six common symptoms stratified by FEV1/FVC. Across the LC13 symptom scales, most scores, except dyspnoea were similar between patients with COPD (FEV1/FVC <70%) and without COPD (FEV1/FVC ≥ 70%) at baseline. There was a clinically significant difference at baseline for dyspnoea, but this difference diminished over time, at Cycle 12, there was no significant difference.

## Discussion and conclusion

In our study population, over 60% of patients had coexisting COPD, placing them at the higher end of the prevalence range reported in other settings (40–70% among NSCLC patients) [6–8]. The COPD group have more squamous cell carcinoma, more patients with a smoking history and more patients in Eastern Cooperative Oncology Group (ECOG) Performance Status 1. Generally, the patients with COPD are more frail, and it is important to learn more about how this impacts the treatment tolerability.

Our results show that there was no significant difference over time in overall global health as assessed through the EORTC QLQ-C30, for patients with locally advanced NSCLC treated with chemoradiation and durvalumab, when looking at the whole group (Figure 2a). This aligns well with a previous study in a similar population [27]. Hui and coworkers reported global health status and quality of life remained stable during treatment with chemoradiation ± durvalumab in the PACIFIC trial [27]. This may indicate that patients tolerate the treatment reasonably well and do not experience a decline in quality of life compared to their baseline level. However, when looking at global health in two groups with different pulmonary function (FEV1/FVC < and ≥ 0.7), we see that the patients with COPD showed a steady improvement in global health across all assessments except at Cycle 12, where a significant decline was observed. This deterioration suggests an increased treatment burden in this vulnerable cohort. The fact that one‑third of these patients are active smokers may further potentiate treatment‑related toxicity, accelerate decline in lung function, and ultimately contribute to poorer quality‑of‑life outcomes.

We identified clinically significant differences in the QLQ-C30 symptoms Fatigue, Pain and Dyspnoea at baseline, however diminishing difference during the treatment timeline. The number of responders is significantly lower at end of treatment as compared to baseline, and the decline in responders might of course influence the results. Patients with the most severe symptoms might not be represented at Cycle 12.

In the QLQ-LC13 symptom scale, no significant group differences were identified, except for dyspnoea at baseline, where the group with a poor pulmonary function reported higher scores, as would be expected. The two groups differ in several aspects, like smoking history and performance status, which might impact the overall well-being of the patients.

A limitation of this study is the relatively small sample size, and challenges in controlling for other factors impacting the HRQoL, which may reduce the statistical power and limit the generalizability of the findings. As expected, compliance with the questionnaires decrease over time, which partly is related to disease progression and deaths. Moreover, missing data on several items, including DLCO, could have offered further insight into the interplay between lung function and HRQoL, representing an important avenue for future research.

For patients with stage III NSCLC, chemoradiation followed by immunotherapy has significantly improved prognosis [4, 5, 19]. Despite the high prevalence of comorbidities such as COPD, our findings indicate that the treatment is generally well tolerated across patient groups. Although patients with impaired lung function reported lower overall global health scores at end of treatment, no substantial differences were observed in specific QLQ-C30 symptom domains between groups, at end of treatment. These results reinforce the safety and efficacy of this treatment approach, even in patients with compromised pulmonary function, and highlight the importance of individualized monitoring and multidisciplinary care to optimize clinical outcomes.

## Data Availability

Data are available upon request.
